# Supplementary material for: YAP/TAZ Promote GLUT1 Expression and Are Associated with Prognosis in Endometrial Cancer
Source: Cancers (Basel). 2025 Aug 1;17(15):2554. doi: 10.3390/cancers17152554 (PMC12345884; doi:10.3390/cancers17152554)
Supplement: Supplementary file 1 [file cancers-17-02554-s001.zip › cancers-3762057-supplementary.pdf]

Supplementary Materials

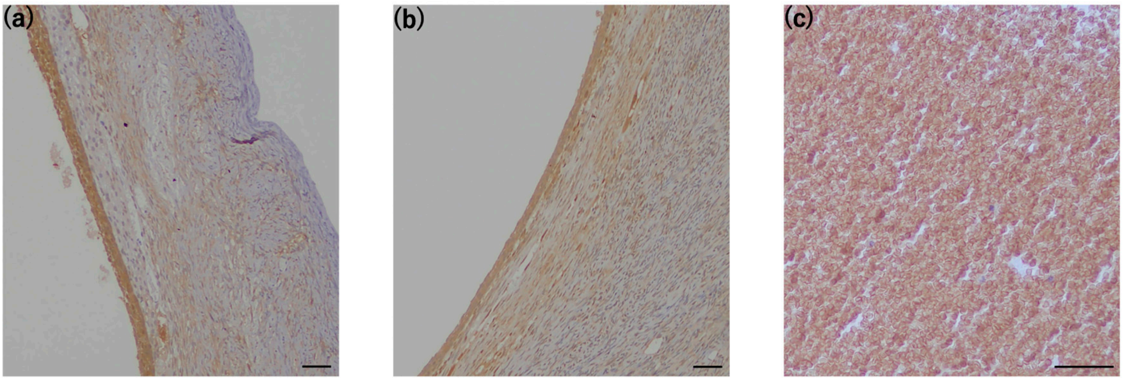

Figure S1. Representative immunostaining of YAP, TAZ, and GLUT1 in ovaries and erythrocytes. (**a**) YAP was strongly expressed in the ovarian corpus luteum (magnification, ×100). (**b**) TAZ was strongly expressed in the ovarian corpus luteum (magnification, ×100). (**c**) GLUT1 was strongly expressed in erythrocytes (magnification, ×200). Scale bars = 50 μm.

Supplementary Table S1. Sequences (sense strand) of siRNAs used in this study.

**Target**	**Sequence (5'-3')**
YAP1	GGUGAUACUAUCAACCAAAdTdT
YAP2	GCACCUAUCACUCUCGAGAdTdT
TAZ1	AGGUACUUCCUCAAUCACAdTdT
TAZ2	GACAUGAGAUCCAUCACUAdTdT
NC	UUCUCCGAACGUGUCACGUdTdT

Supplementary Table S2. Sequences of primers used in RT-qPCR analyses.

**Nucleotide Sequence (5′-3′)**	
SLC2A1 F	ATACTCATGACCATCGCGCTAG
SLC2A1 R	AAAGAAGGCCACAAAGCCAAAG
CYR61 F	CCTCGGCTGGTCAAAGTTAC
CYR61 R	TTTCTCGTCAACTCCACCT
YAP F	GCAGTTGGGAGCTGTTTCTC
YAP R	CTGTCGAAGATGCTGAGCTG
TAZ F	GGCTGGGAGATGACCTTCAC
TAZ R	CTGAGTGGGGTGGTTCTGCT
36B4 F	GCTGCAGCCCCAGCTAAGGT
36B4 R	TAAGTTGGTTGCTTTTTGGT
